# Protection of nontarget structures in prostatic artery embolization

**DOI:** 10.1590/0100-3984.2021.0021

**Published:** 2022

**Authors:** Bruna Ferreira Pilan, André Moreira de Assis, Airton Mota Moreira, Vanessa Cristina de Paula Rodrigues, Francisco Cesar Carnevale

**Affiliations:** Radiology Department, Faculdade de Medicina da Universidade de São Paulo (FMUSP), São Paulo, SP, Brazil.

**Keywords:** Prostate, Prostatic hyperplasia, Embolization, therapeutic/methods, Erectile dysfunction, Próstata, Hiperplasia prostática, Embolização terapêutica/métodos, Disfunção erétil

## Abstract

**Objective:**

To describe the efficacy and safety of protective embolization during prostatic artery embolization, as well as to discuss its clinical relevance.

**Materials and Methods:**

This was a retrospective, observational, single-center study including 39 patients who underwent prostatic artery embolization to treat lower urinary tract symptoms related to benign prostatic hyperplasia between June 2008 and March 2018. Follow-up evaluations, performed at 3 and 12 months after the procedure, included determination of the International Prostate Symptom Score, a quality of life score, and prostate-specific antigen levels, as well as ultrasound, magnetic resonance imaging, and uroflowmetry.

**Results:**

Protective embolization was performed in 45 arteries: in the middle rectal artery in 19 (42.2%); in the accessory internal pudendal artery in 11 (24.4%); in an internal pudendal artery anastomosis in 10 (22.2%); in the superior vesical artery in four (8.9%); and in the obturator artery in one (2.2%). There was one case of nontarget embolization leading to a penile ulcer, which was attributed to reflux of microspheres to an unprotected artery. There were no complications related to the protected branches. All of the patients showed significant improvement in all of the outcomes studied (*p* < 0.05), and none reported worsening of sexual function during follow-up.

**Conclusion:**

Protective embolization can reduce nontarget embolization during prostatic artery embolization without affecting the results of the procedure. In addition, no adverse events other than those expected or previously reported were observed. Therefore, protective embolization of pudendal region is safe.

## Abstract (pt)

**Objetivo:**

Descrever a eficácia e a segurança da embolização de proteção na embolização de artérias prostáticas e discutir sua relevância clínica.

**Materiais e Métodos:**

Estudo retrospectivo, observacional, de um único centro, que inclui 39 pacientes submetidos a embolização de artérias prostáticas para tratamento de sintomas do trato urinário inferior relacionados a hiperplasia benigna da próstata, de junho de 2008 a março de 2018. O acompanhamento foi realizado em 3 meses e 12 meses, incluindo International Prostate Symptom Score, escore de qualidade de vida, antígeno prostático específico, ultrassom, ressonância magnética e urofluxometria.

**Resultados:**

Embolização de proteção foi realizada em 45 artérias: artérias retais médias em 19 (42,2%); artérias pudendas internas acessórias em 11 (24,4%); anastomoses com ramos da artéria pudenda interna em 10 (22,2%); artérias vesicais superiores em quatro (8,9%); e artéria obturatória em uma (2,2%). Houve um caso de embolização não alvo que provocou uma úlcera peniana, atribuída a refluxo de partículas para uma artéria não protegida. Não houve complicações relacionadas com os ramos protegidos. Os pacientes apresentaram melhora significativa em todos os resultados estudados (*p* < 0,05) e não relataram piora da função sexual durante o acompanhamento.

**Conclusão:**

Embolização de proteção pode ser realizada para diminuir embolização não alvo sem interferir nos resultados da embolização de artérias prostáticas. Além disso, não foi observado nenhum evento adverso diferente dos já esperados ou previamente publicados. A embolização de proteção na região pudenda é segura.

## INTRODUCTION

Prostatic artery embolization (PAE) as a treatment for benign prostatic hyperplasia (BPH) was first reported by DeMeritt et al.^(1)^ in a patient with refractory hematuria. Carnevale et al.^(2)^ were the first to use PAE successfully for the treatment of lower urinary tract symptoms (LUTS) due to BPH, thus showing it to be a viable treatment alternative. Since then, studies have established PAE as a safe, effective treatment, showing it to be associated with a significant reduction in prostate size and in elasticity, which leads to better functional and clinical outcomes(^3^,^4^,^5^,^6^,^7^).

The vascular anatomy of the prostate has been described in angiography and cadaver studies(^8^,^9^,^10^,^11^). Blood is supplied to the prostate mainly by two branches of the prostate artery: an anteromedial branch, which irrigates the transition zone of the prostate gland; and a posterolateral branch, which irrigates the apex and peripheral zone. Relevant arterial anastomoses typically involve the posterolateral branch. Knowledge of the vascular anatomy of the prostate and its variations, as well as a meticulous analysis during the procedure, is crucial because misinterpretation of the anatomy could result in nontarget embolization (NTE) of periprostatic organs and structures such as the bladder, rectum, and penis^(12)^. Protective embolization (PE) of nontarget arteries or extraprostatic anastomoses, typically using coils or gelatin sponges, can be performed to redirect blood flow to the prostatic artery and avoid NTE. However, due to the terminal nature of the irrigation of structures such as the penis, rectum, and bladder, there is a theoretical risk of distal ischemia when PE is used. In addition, the impact that PE of the internal pudendal artery branches and the accessory internal pudendal artery has on sexual function is a matter of concern.

Because PAE to treat LUTS attributed to BPH is a relatively recent technique, there have been few studies of its efficacy and safety, even fewer addressing specific issues like PE. There have also been few reports of coil embolization during PAE. Two of those reports were single-center studies including a small number of patients(^13^,^14^), and the rest were case reports(^15^,^16^,^17^,^18^,^19^). The present study expands the literature by describing a single-center experience of the efficacy and safety of PE during PAE. We also discuss the technical aspects of the procedure and the clinical relevance of the findings.

## MATERIALS AND METHODS

Between June 2008 and March 2018, a total of 305 patients underwent PAE at a tertiary hospital in Brazil. This was a retrospective, observational, single-center study, including the 39 patients who underwent PAE in that period and required PE to avoid NTE. The institutional review board approved the study, and all participating patients gave written informed consent. All procedures were performed in accordance with the standards established by the local research ethics committee and in the Declaration of Helsinki. This manuscript was composed in accordance with the Strengthening the Reporting of Observational Studies in Epidemiology statement.

The indications for PAE were moderate to severe LUTS—defined as those resulting in an International Prostate Symptom Score (IPSS) > 7—with failure or intolerance of pharmacological treatment (alpha-blockers, 5-alpha reductase inhibitors, or both), and refusal of or contraindication to surgical treatment. Patients with large bladder diverticulum were excluded, as were those with bladder stones, obstructive chronic kidney disease, urethral stenosis, neurogenic bladder, or prostate cancer.

Follow-up evaluations were performed at 3 and 12 months after PAE, being performed annually thereafter. At each evaluation, the IPSS questionnaire was applied and a quality of life (QoL) score was determined, as was the prostate-specific antigen (PSA) level; patients also underwent uroflowmetry, ultrasound, and magnetic resonance imaging (MRI). All MRI examinations were performed by the same, experienced radiologist. As measured on MRI, the prostate volume (PV) was calculated (in cm ) by using the ellipsoid formula:

PV=[cephalocaudal, transverse, and anteroposterior diameters×π/6]

Clinical and urological outcomes, as well as adverse event data, were obtained systematically in previously scheduled medical appointments during follow-up. During those consultations, sexual function was evaluated subjectively (i.e., no specific sexual function scale was applied).

### Technical protocol for PAE

The PAE was performed under local anesthesia, at a day hospital, by three different interventional radiologists, all of whom were experienced in performing the procedure. Vascular access was obtained by puncture of the right common femoral artery through a 5-Fr introducer sheath. Selective catheterization of prostatic arteries was performed with 2.4-Fr or smaller microcatheters (Progreat; Terumo, Tokyo, Japan). A guide wire (PT2 [0.014*"*] or Fathom [0.016*"*]; Boston Scientific, Marlborough, MA, USA) was used in order to push and deploy the coils.

Ipsilateral oblique incidences ranging from 20° to 50° were used for the identification and catheterization of the prostatic arteries. When necessary to confirm the findings of the angiography, cone-beam computed tomography was performed with contrast (3–5 mL, administered by power injection at 0.3 mL/sec), with a 5-s spin (40°/s) and a 10-s delay. Trisacryl gelatin microspheres (Embosphere; Merit Medical Systems, South Jordan, UT, USA), ranging from 100–500 µm in diameter, were used for embolization. From 2008 to 2016, 300–500 µm microspheres were used, in accordance with the efficacy and safety studies available at the time(^2^,^3^,^4^,^5^). However, since December 2016, a combination of 100 300 µm and 300 500 µm microspheres has been used, in an effort to reduce the recurrence of LUTS after PAE. The choice of particle size was therefore not influenced by anatomical or vascular factors.

Immediately before embolization, digital subtraction angiography, with hand injection of contrast medium, was performed in order to simulate embolization conditions following intra-arterial administration of a vasodilator (isosorbide mononitrate). The PE was performed in accordance with the following criteria: the presence of a high-flow anastomosis (with a retrograde flow pattern in relation to that of the prostate, detected even during slow, hand injection of contrast medium) to a clinically relevant territory (penis, bladder, or rectum); or reflux to clinically relevant arterial segment (middle rectal, internal pudendal, accessory internal pudendal, or bladder branches). The PE was performed with 0.018*"* coils (VortX; Boston Scientific) or with gelatin sponges (Gelita-Spon; Gelita Medical, Eberbach, Germany). The PAE was then performed with microspheres until total stasis had been achieved, as previously described^(20)^. The safety of PE was assessed according to the Clavien-Dindo classification of surgical complications^(21)^, adapted to PAE.

All statistical tests were performed with GraphPad Prism software, version 3.0 (GraphPad Software Inc., San Diego, CA, USA). Baseline and follow-up values for the IPSS, QoL score, peak urinary flow rate (Q_max_, obtained with uroflowmetry), P V, PSA level, and post-void residual (PVR) volume are expressed as mean ± standard deviation (SD). For those same variables, the change after PAE is expressed as mean and 95% confidence interval (95% CI). Values were compared between time points using paired t-tests. The significance level for all statistical tests was defined as a two-tailed *p*-value of 0.05 or less.

## RESULTS

Among the 305 patients who underwent PAE during the study period, extraprostatic anastomosis or reflux with potential for NTE was identified in 39 (12.8%), with a total of 45 occluded arteries (Table 1). Of those 39 patients, 23 (59.0%) underwent PAE with 300–500 µm microspheres, whereas 16 (41.0%) underwent PAE with 100–300 µm and 300–500 µm microspheres. All 39 patients showed significant improvement of LUTS after PAE, the mean changes being as follows: a reduction of 18.9 ± 7.1 in the IPSS, a reduction of 3.4 ± 1.5 in the QoL score, an increase of 8.8 ± 5.9 mL/s in the Qmax, a reduction of 31.8 ± 24.0 cm^3^ in P V, a reduction of 2.5 ± 3.9 ng/mL in the PSA level, and a reduction of 119.1 ± 197.3 mL in the PVR volume (*p* < 0.01 for all). Baseline and 12-month follow-up data are summarized in Table 2.

**Table:** Details of PE performed during PAE of occluded arteries (N = 45).

Protected region	Arterial segment embolized	n (%)
Rectal	L MRA	8 (17.8)
	R MRA	11 (24.5)
Pudendal	L AM–R IPA anastomosis	1 (2.2)
	R PL–R IPA anastomosis	1 (2.2)
	L PL–L IPA anastomosis	1 (2.2)
	R AM–R IPA anastomosis	3 (6.6)
	L AM–L IPA anastomosis	1 (2.2)
	L AM–L and R IPA anastomosis	2 (4.4)
	R AM–L IPA anastomosis	1 (2.2)
	R aIPA	5 (11.1)
	L aIPA	6 (13.3)
Bladder	L AM–L and R SVA	3 (6.6)
	L AM–R SVA	1 (2.2)
Obturator	R AM–L and R ObtA	1 (2.2)

L, left; R, right; MRA, middle rectal artery; AM, anteromedial (branch of the prostatic artery); IPA, internal pudendal artery; PL, posterolateral (branch of the prostatic artery); aIPA, accessory internal pudendal artery; SVA, superior vesical artery; ObtA, obturator artery.

**Figure 1:**
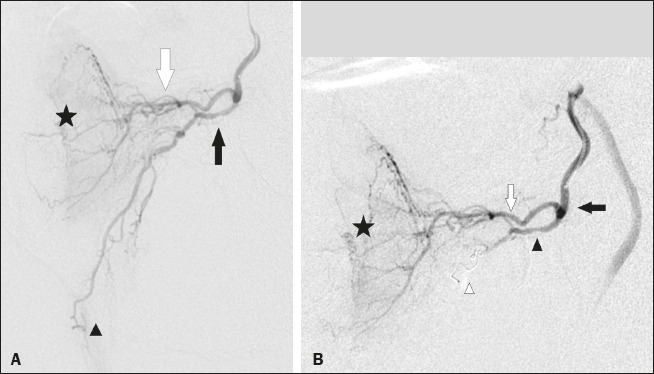
A: Selective digital subtraction angiography of the left prostatic artery, ipsilateral oblique view. White arrow: anteromedial branch; black arrow: common trunk of the posterolateral branch of the prostatic artery and middle rectal artery; arrowhead: middle rectal artery; star: prostate gland. B: Selective prostatic artery digital subtraction angiography after PE of the posterolateral branch-rectal trunk. Black arrow: prostatic artery; white arrow: anteromedial branch; black arrowhead: posterolateral branch-rectal trunk; star: prostate gland; white arrowhead: microcoil.

**Figure 2:**
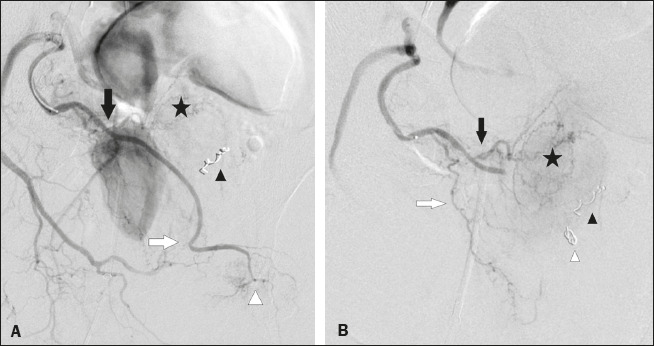
**A:** Selective digital subtraction angiography of the accessory pudendal artery. Black arrow: prostatic branch; white arrow: distal accessory internal pudendal artery; star: prostate gland; white arrowhead: pudendal territory; black arrowhead: protective coil embolization of the contralateral middle rectal artery. **B:** Digital subtraction angiography after PE of the distal accessory internal pudendal artery. Black arrow: anteromedial prostatic branch; white arrow: posterolateral branch of the prostatic artery; star: prostate gland; white arrowhead: protective coil embolization of an accessory internal pudendal artery; black arrowhead: protective coil embolization of the contralateral middle rectal artery.

Microcoils were deployed in the middle rectal artery (Figure 1) in 19 (42.2%) of the 45 cases of occluded arteries; in the accessory internal pudendal artery (Figure 2) in 11 (24.4%); in an internal pudendal artery anastomosis in 10 (22.2%); in the distal superior vesical artery in four (8.9%); and in the distal obturator artery in one (2.2%). A total of 61 microcoils (3.0 × 3.3 mm, 3.0 × 2.5 mm, 4.0 × 3.7 mm, or 2.0 × 3.0 mm) were deployed and, in one case, a gelatin sponge was used. Bilateral PE was necessary in five (12.8%) of the 39 patients. In one patient (2.5%), PE was performed for two arteries on the same side of the pelvis. Another patient who underwent PE developed a glans penis ulcer in the second week after the procedure. The ulcer healed within 30 days with local treatment. Although that patient had undergone technically successful PE in the left accessory internal pudendal artery, there was reflux of a significant amount of 300–500 µm microspheres into the internal pudendal artery during embolization of the right side of the prostate, leading to NTE. None of the patients reported worsening of sexual function during the follow-up period.

## DISCUSSION

For patients with BPH-related LUTS, PAE has been used as an alternative treatment with the aim of reducing prostate size and improving elasticity, thus providing symptom relief(^2^,^3^,^4^,^5^,^6^,^7^). Although there is currently sufficient evidence that PAE is a safe procedure, NTE is still a concern and several cases of NTE-related complications have been described. Moreira et al.^(22)^ reported a case of a transient ischemic rectitis, in which rectal ulcers that were identified on colonoscopy disappeared in two weeks without treatment. In one recent review of the literature^(23)^, the reported incidence of transient rectal bleeding after PAE was found to range from 2.4% to 27.0%. Bilhim et al.^(24)^ reported a 7% incidence of adverse events affecting the penis (small ischemic skin lesions or transient erectile dysfunction) after PAE. In the affected patients, a penile shunt was retrospectively identified on angiography after selective positioning of the microcatheter prior to embolization. If PE had been performed, the penile adverse events observed in those cases might have been avoided. Finally, Pisco et al.^(5)^ reported a case of post-PAE bladder wall ischemia that required surgical repair.

Although the arterial blood supply to the pelvis is widely interconnected by anastomoses, most of them are characterized by low flow, being identifiable on arteriogram or cone-beam computed tomography with power injection of contrast medium, with or without injection of a vasodilator, and usually do not require PE. In addition, migration of small amounts of embolic agents through an anastomosis involving the obturator region or other pelvic parietal structures may not lead to clinically relevant complications and it might therefore be unnecessary to perform PE in such cases^(11)^. Furthermore, PE with microcoils or gelatin sponges can reduce the risk of NTE in cases of high-flow anastomosis, as well as being capable of preventing distal reflux of embolic agents to clinically relevant regions(^12^,^13^). Extraprostatic anastomosis and reflux were the indications for PE in the present study. Moreover, PE can redirect particle flow during PAE^(14)^ and maintains adequate distal perfusion of nontarget structures.

**Table:** Values at baseline and at 12 months after PAE with PE.

Variable	Baseline Mean ± SD	At 12 months of follow-up Mean ± SD	Change Mean (95% CI)	*P*
IPSS	22.7 ± 5.53	3.46 ± 3.41	-18.91 (-21.81;-16.01)	< 0.001
QoL score	4.84 ± 0.90	1.37 ± 0.90	-3.37 (-3.99; -2.74)	< 0.001
Qmax (mL/s)	7.14 ± 4.10	14.27 ± 7.06	8.77 (6.19; 11.35)	< 0.001
PV (cm^3^)	96.63 ± 51.22	70.56 ± 28.59	-31.81 (-42.60; -21.01)	< 0.001
PSA (ng/mL)	4.31 ± 3.10	2.26 ± 1.31	-2.51 (-4.20; -0.83)	0.010
PVR volume (mL)	123.67 ± 169.30	34.56 ± 23.87	-119.10 (-210.27; -27.93)	0.024

In a study of 122 patients who underwent PAE, Bhatia et al.^(14)^ reported that coil embolization was required for 39 arteries in 32 (26.2%) of the patients. Among those 39 arteries, coil embolization was employed to avoid NTE in 36, to treat prostatic artery extravasation in two, and to occlude an intraprostatic arteriovenous fistula in one. The authors compared the two groups of patients who underwent PAE: coil-embolization and no-coil-embolization. The level of radiation exposure (dose-area product) was higher in the coil-embolization group than in the no-coilembolization group, although the difference was not significant, whereas the procedure and fluoroscopy times were significantly longer in the former group. There was one major complication (urosepsis) in each group, as well as one minor ischemic complication in the coil-embolization group, the affected patient requiring bilateral embolization of the internal pudendal artery to treat prostatic artery extravasation after embolization. There were no significant differences between the groups regarding major and minor complications at 1 and 3 months of follow-up, nor were there any reports of erectile dysfunction. In another study, involving 55 patients, Amouyal et al.^(13)^ evaluated the safety and efficacy of 11 shunt exclusions followed by PAE, in comparison with 44 cases in which PE was not employed. In that study, PE was performed in 20% of the patients. Among the 11 cases in which PE was required, the indication was a penile shunt in seven (64%), a rectal shunt in two (18%), and the penile shunt-rectal shunt combination in two (18%). None of the patients developed skin complications (ulcer, necrosis, edema, or redness) or showed a decrease in their total International Index of Erectile Function (IIEF) score.

**Figure 3:**
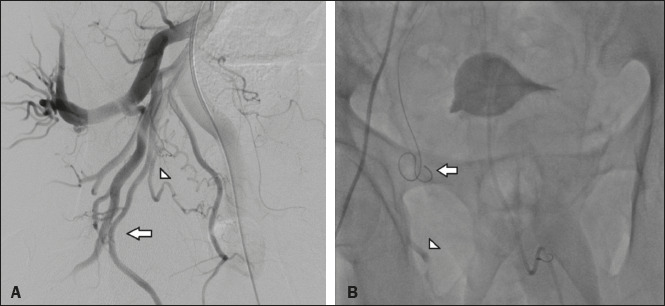
**A:** Selective digital subtraction angiography of the right internal iliac artery, ipsilateral oblique view. The prostatic artery (arrowhead) originates from the internal pudendal artery—representing a type IV variation (arrow)—in a very short trunk. **B:** Fluoroscopy without digital subtraction angiography or contrast injection, after PAE. The microcatheter is within the prostatic artery (arrow). Note the low flow of the contrast medium in the internal pudendal artery (arrowhead), indicating that there was reflux of the microspheres, which was the cause of the NTE in this patient.

In our sample, there was one patient who had an ischemic complication, which was unrelated to the PE. In that patient, embolization of the left accessory internal pudendal artery was performed successfully with a metallic coil. However, in the contralateral side of the pelvis, the prostatic artery originated from the internal pudendal artery in a very short trunk (Figure 3A). A retrospective review of the images indicated that the NTE was likely caused by reflux of microspheres to the pudendal region in the right side of the pelvis (Figure 3B), rather than by the PE procedure. Therefore, the failure in that case was that the high risk of particle reflux to a relevant nontarget artery was not identified. That is in keeping with the results of previous studies of PE in PAE(^13^,^14^), in which it was reported that there were no adverse events or significant differences between PAE requiring PE and PAE not requiring PE in terms of the rates of adverse events related to PE. It is noteworthy that the proportion of patients submitted to PE in the present study (12.8%) was lower than the 26.2% and 20.0% reported by Bhatia et al.^(14)^ and Amouyal et al.^(13)^, respectively. One hypothesis to explain the lower use of PE in our sample is that the microcath-eter was placed more distally in the prostatic artery, in an attempt to perform the technique in which distal embolization is performed after proximal embolization^(20)^.

The PE technique has some pitfalls, such as malpositioning of the protective embolic agent and failure to achieve complete thrombosis of the nontarget artery or anastomosis. In our sample, coils were the embolic agents of choice, because of their precision of deployment and lower risk of microcatheter occlusion. Because of the long, straight courses of the branches to be embolized and the high cost of detachable microcoils, the more affordable, pushable coils were used. In one patient, a gelatin sponge was used because no microcoil of an appropriate size was available. In another study, the protective embolic agent of choice for the pudendal region was a gelatin sponge, which was considered a temporary embolic agent^(13)^. The positioning of the embolic agent is also important: when placed too distally, revascularization paths could be excluded, which could itself lead to NTE. Conversely, when the embolic agent is placed too proximally, prostatic artery branches can be occluded, blocking the deployment of microspheres to the prostatic vascular bed, mainly to the apex to the prostate^(14)^. Positioning the protective material appropriately can be challenging because of the need for distal navigation through tortuous branches with atherosclerotic plaques. However, in the present study, no dissection or significant vasospasm was seen and PE was feasible whenever necessary. The use of microcatheters and delicate microwires played an important role in that it enabled catheterization of the more distal branches.

The size of the particles can also have an effect on NTE. Smaller microspheres could penetrate more distally, causing more ischemia and necrosis. Although that could lead to greater prostate infarction, prostate reduction, and clinical improvement, it could also increase the incidence of adverse events affecting nontarget structures if the microspheres pass through small distal microshunts(^25^,^26^,^27^,^28^). In some studies, the rate of minor adverse events has been reported to be higher in patients who undergo PAE with smaller microspheres, although the difference was not statistically significant(^29^,^30^,^31^). In the present study, the NTE in the PE group occurred when 300–500 µm microspheres were used and was related to reflux of the embolic agent.

Erectile dysfunction is one of the major causes of concern when PE is necessary, especially when the targets are internal pudendal artery branches or the accessory internal pudendal artery. Experimental studies have shown that, after unilateral acute clamping of the internal pudendal artery, there is compensatory contralateral flow, with moderate impairment of the intracavernous pressure. Bilateral occlusions have been shown to result in a marked reduction in the intracavernous pressure and a minimal response to cavernous nerve stimulation^(32)^. Recent anatomical studies have described an intricate network of blood vessels responsible for the penile blood supply, in which the accessory internal pudendal artery also plays a critical role^(33)^. However, in a prospective study of 200 patients undergoing radical prostatectomy, Box et al.^(34)^ reported preserved erectile function in 95% of patients in whom an accessory internal pudendal artery was sacrificed. Those authors also found that the sacrifice of one accessory internal pudendal artery did not correlate significantly with the time to erectile function recovery, quality of postoperative erections, or mean IIEF score. It is possible that unilateral embolization of internal pudendal artery branches or an accessory internal pudendal artery is safe regarding sexual function, given that contralateral flow is preserved, although that is still a matter of debate. In the present study, 10 (25.6%) of the 39 patients underwent PE of an accessory internal pudendal artery and 10 (25.6%) underwent PE of an internal pudendal artery anastomosis; none of those patients reported sexual impairment after PAE. In other studies of PE, there have also been no reports of development or worsening of erectile dysfunction(^13^,^14^,^15^). In fact, the authors of recent studies have encouraged the use of PE whenever the penile blood supply is involved(^12^,^13^,^14^,^18^,^19^,^35^).

The clinical, biochemical, imaging, and urodynamic outcomes in our sample were in line with data previously reported for patients undergoing PAE(^1^,^2^,^3^,^4^,^5^,^6^,^7^,^36^,^37^,^38^). All of the patients in our sample showed statistically significant improvements in all of the parameters analyzed. These results suggest that PE does not negatively affect the results of PAE, possibly because of the very distal navigation into the anastomosis during PE, which prevented blockage of prostatic artery branches. This technical aspect is critical and should be taken into consideration during the procedure.

Our study has some limitations, especially in relation to the small sample size and the single-center, retrospective nature of the design. No pre- or post-PAE IIEF scores were available. Nevertheless, there were no reports of erectile dysfunction in our sample. Although PAE has proven to be safe and effective, there is a need for further studies aimed at optimizing the technical aspects.

## CONCLUSION

The use of PE can reduce the risk of NTE during PAE without affecting the results of the procedure. In addition, PE does not appear to increase the risk of adverse events after PAE. Our findings indicate that PE of the pudendal region is safe, resulting in no significant changes in erectile function. Knowledge of the anatomical aspects of the prostate is paramount for achieving optimal results.
